# O_2_ Carrier Myoglobin Also Exhibits β-Lactamase Activity That Is Regulated by the Heme Coordination State

**DOI:** 10.3390/molecules27238478

**Published:** 2022-12-02

**Authors:** Shuai Tang, Ai-Qun Pan, Xiao-Juan Wang, Shu-Qin Gao, Xiang-Shi Tan, Ying-Wu Lin

**Affiliations:** 1 School of Chemistry and Chemical Engineering, University of South China, Hengyang 421001, China; 2 Lab of Protein Structure and Function, University of South China Medical School, Hengyang 421001, China; 3 Department of Chemistry and Institute of Biomedical Science, Fudan University, Shanghai 200433, China

**Keywords:** heme protein, myoglobin, β-lactamase, ampicillin, heme coordination

## Abstract

Heme proteins perform a variety of biological functions and also play significant roles in the field of bio-catalysis. The β-lactamase activity of heme proteins has rarely been reported. Herein, we found, for the first time, that myoglobin (Mb), an O_2_ carrier, also exhibits novel β-lactamase activity by catalyzing the hydrolysis of ampicillin. The catalytic proficiency ((*k*_cat_/*K*_M_)/*k*_uncat_) was determined to be 6.25 × 10^10^, which is much higher than the proficiency reported for designed metalloenzymes, although it is lower than that of natural β-lactamases. Moreover, we found that this activity could be regulated by an engineered disulfide bond, such as Cys46-Cys61 in F46C/L61C Mb or by the addition of imidazole to directly coordinate to the heme center. These results indicate that the heme active site is responsible for the β-lactamase activity of Mb. Therefore, the study suggests the potential of heme proteins acting as β-lactamases, which broadens the diversity of their catalytic functions.

## 1. Introduction

Since the discovery of penicillin, the types of antibiotics have increased dramatically, and they have been widely used in medicine, animal husbandry and aquaculture [1,2]. Antibiotics are easy to produce but difficult to remove from the environment, which causes pathogenic microorganisms to develop antibiotic resistance. Therefore, it is urgent to solve the problem of antibiotic resistance globally [3,4,5]. As one family of antibiotics, β-lactam antibiotics are widely used because of their high efficiency, accounting for 50–70% of the total consumption of antibiotics [6,7], and the global use of ampicillin (AMP) is growing each year [8,9].

Research on the degradation of β-lactam antibiotics is developing rapidly. To date, various chemical methods have been reported for the degradation of β-lactam antibiotics, such as hydrolysis [10,11,12], oxidation [13] and photolysis. [14]. Compared to the traditional chemical methods, enzymatic catalysis provides a gentle method for the degradation of β-lactam antibiotics. β-lactamases were first discovered in *Staphylococcus aureus* strains, which were classified as A–D [15,16,17].

**Figure 1:**
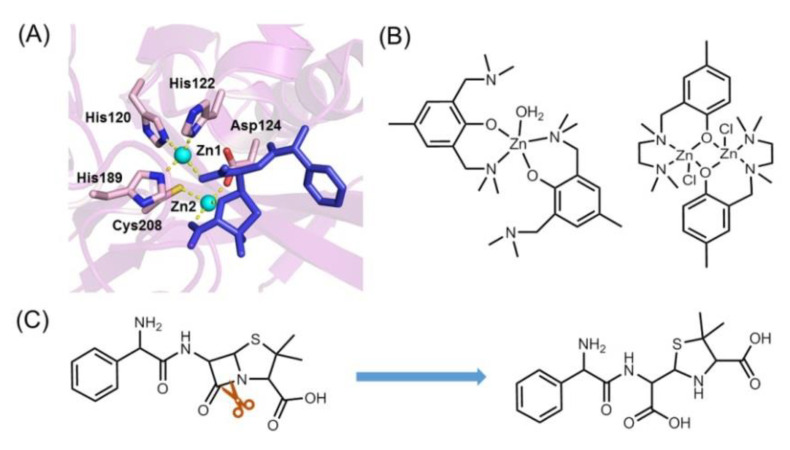
(**A**) The X-ray structure of New Delhi metallo-β-lactamase in complex with hydrolyzed AMP (PDB code 6OL8 [18]), showing the active coordination site and the binding of AMP. (**B**) Representative model complexes for metallo-β-lactamase [22]. (**C**) Chemical structure of AMP and the hydrolytic reaction by cleavage of the β-lactam ring.

Classes A, C and D use serine residues as the active site (namely, serine β-lactamase), whereas class B contains one or two Zn^2+^ or other metal ions (namely, metallo-β-lactamase), for example, the New Delhi metallo-β-lactamase (NDM) (Figure 1A) [18]. Moreover, various metal complexes were designed to mimic the active site of metallo-β-lactamase (Figure 1B) [19].

In addition to model metal complexes, artificial enzymes were designed to exhibit lactamase activity. For example, Song and Tezcan presented a designed supramolecular protein assembly with β-lactamase activity. Furthermore, other metalloenzymes may also exhibit lactamase activity, such as pig liver carboxylesterase, which exhibits high degradation activity toward AMP [20,21].

**Figure 2:**
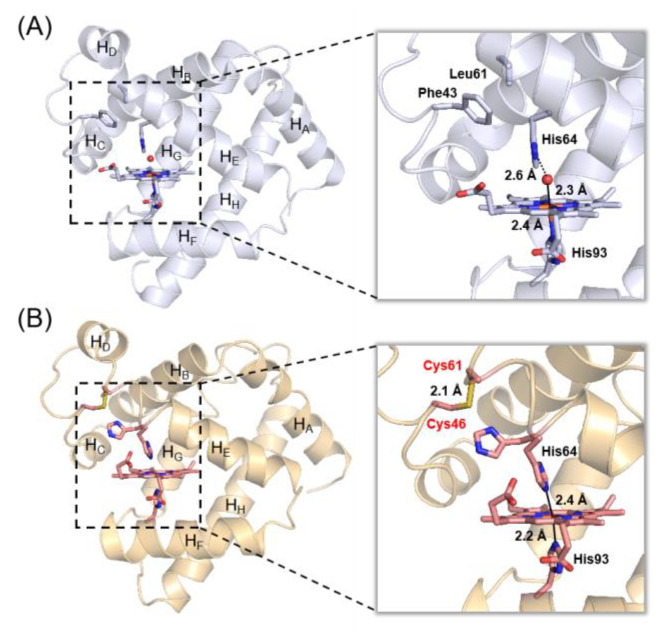
X-ray structure of WT Mb (PDB code 1JP6 [43], **A**) and F46C/L61C Mb (PDB code 7VW4 [44], **B**), showing the overall structure (left) and the heme coordination site (right), respectively.

Heme proteins are a large class of metalloproteins, and they perform many vital biological functions, of which the role of the heme iron center is crucial [23,24,25,26]. For example, as an O_2_ carrier, myoglobin (Mb) has a six-coordination heme (H_2_O/His93) with a distal His64 (Figure 2A), and, as an electron transfer protein, cytochrome *c* (Cyt *c*) has a heme center with a six-coordination of Met/His [27,28]. 

As an ideal model for protein design, Mb has been applied to construct functional enzymes, such as those capable of reduction of O_2_/NO/NO_2_^−^ and the oxidation of organic compounds [29,30,31,32,33,34,35,36]. Moreover, Mb has been designed to catalyze carbene transfer reactions and others by modifying the heme center, heme replacement and directed evolution [37,38,39,40,41,42]. These achievements provide valuable insights into the relationship between the structure and function of heme proteins.

Recently, to regulate the structure and function of Mb, we have designed a disulfide bond in Mb by introducing a couple of Cys residues at positions 46 and 61 and found that the double mutant adopts F46C/L61C Mb both an open heme conformation and a bis-His coordination (His64/His93) with ~50% of each conformation (Figure 2B) [44]. Alternatively, direct titration studies indicated that the imidazole (Im) ligand may convert the heme coordination from H_2_O/His93 to Im/His93 [45]. In a previous study, we demonstrated that the hydrogen-bonding network in the heme active site may regulate the hydrolysis activity of Mb [46]. Therefore, we are interested in testing whether Mb exhibits β-lactamase activity and whether it could be regulated by the heme coordination state.

As shown in this study, we reveal that wild-type (WT) sperm whale Mb can catalyze the hydrolysis of AMP, which could be regulated by the engineered disulfide bond of Cys46-Cys61 in F46C/L61C Mb or by the addition of Im to directly coordinate to the heme center. This study broadens the functional diversity of Mb and suggests that it can potentially act as a β-lactamase.

## 2. Results and Discussion

### 2.1. Hydrolysis of AMP Catalyzed by WT Mb

**Figure 3:**
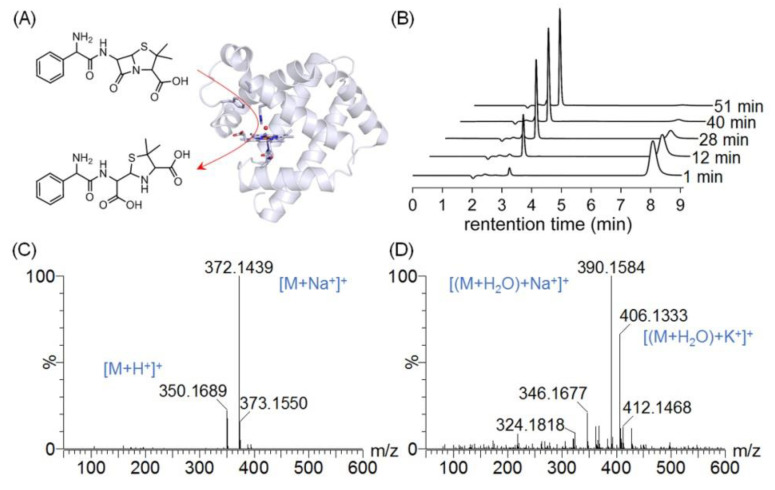
(**A**) Mb-catalyzed hydrolysis of AMP. (**B**) Representative time−dependent HPLC traces, displaying the consumption of AMP catalyzed by WT Mb. (**C**,**D**) ESI-MS spectra of AMP and its hydrolysis product catalyzed by WT Mb: (**C**) AMP (calculated, 349 Da; observed, 350 Da, [M + H^+^]^+^; 372 Da, [M + Na^+^]^+^). (**D**) The hydrolysis product (calculated, 367 Da (M + H_2_O); observed, 390 Da, [(M + H_2_O) + Na^+^]^+^; 406 Da, [(M + H_2_O) + K^+^]^+^).

We first examined the hydrolysis of AMP (Figure 3A) with the addition of WT Mb, which was accomplished by performing the high-performance liquid chromatography (HPLC) analysis of the reaction mixture (Figure 3B) and determining the hydrolysis products by electrospray ionization mass spectrometry (ESI-MS) (Figure 3C,D). As shown in Figure 3B, the HPLC results revealed the freshly prepared AMP and its hydrolysis product with retention times (RTs) of ~7.8 and ~3.2 min, respectively. 

Moreover, the ESI-MS spectra confirmed the conversion from AMP (Figure 3C) to its hydrolysis product (Figure 3D). AMP was presented in forms associated with protons (*m*/*z* = 350, [M + H^+^]^+^) and sodium ions (*m*/*z* = 372, [M + Na^+^]^+^), and its hydrolysis product was represented in forms associated with sodium (*m*/*z* = 390, [(M + H_2_O) + Na^+^]^+^ or potassium ions (*m*/*z* = 406 [(M + H_2_O) + K^+^]^+^, respectively, which agree well with the previous observations [20].

### 2.2. Standard Curve and Self-Hydrolysis of AMP

**Figure 4:**
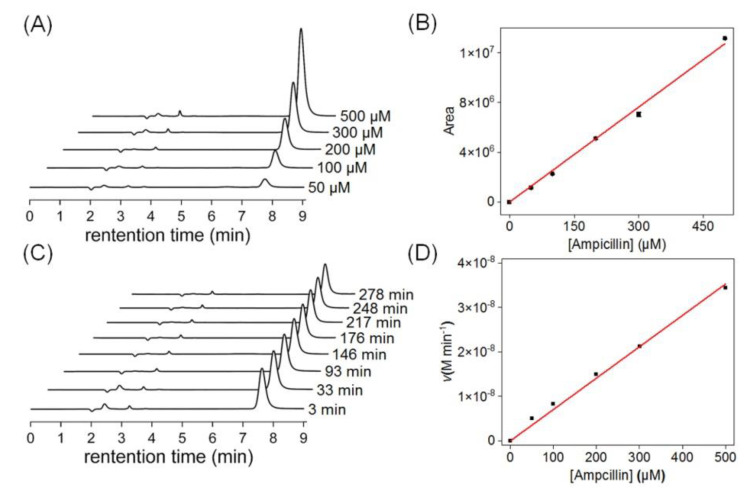
(**A**) A representative set of time−dependent HPLC traces, showing the peak area of AMP at various substrate concentrations. (**B**) Standard curves of AMP measured by HPLC at different concentration gradients. The data are the mean ± SD. (**C**) A representative set of time−dependent HPLC traces, showing the degradation of AMP in 20 mM potassium phosphate buffer (pH 7.0). (**D**) The degradation rate versus concentration of AMP in the same buffer.

Next, we performed HPLC quantitative determination of the standard curve of AMP and its self-hydrolysis in 20 mM potassium phosphate buffer (pH 7.0). As shown in Figure 4A, we measured the peak area of freshly prepared AMP with different concentrations and then obtained its slope by linear fitting The value of the self-degradation rate constant obtained by linear fitting is *k* = 25,482.30 μM^−1^. We further measured the degradation rate of AMP in the same buffer (Figure 4B), and the rate of AMP consumption was measured over ~4.6 h with various substrate concentrations under identical conditions (buffer composition and temperature) to the previous study. This showed that the hydrolysis of AMP in buffer exhibited a rate as low as *k*_uncat_ = 7.06 × 10^−5^ min^−1^.

### 2.3. The β-Lactamase Activity of Mbs and Effects of Im Binding

**Figure 5:**
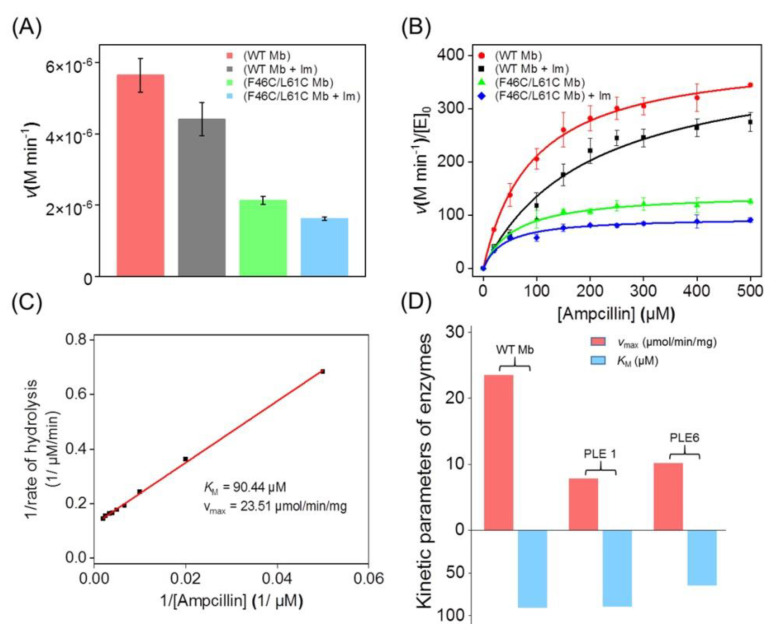
(**A**) Comparison of the β-lactamase activity of WT Mb and F46C/L61C Mb (20 nM) toward AMP (200 μM) with or without the addition of Im (400 nM). (**B**) Michaelis–Menten kinetics of WT Mb and F46C/L61C Mb with or without the addition of Im for AMP hydrolysis. The data are the mean ± SD. (**C**) Hydrolysis of AMP by WT Mb and determination of the enzyme kinetics. Lineweaver–Burk plot of AMP hydrolysis by WT Mb. The *Y*-axis intercept is equal to 1/*v*_max_, and the *X*-axis intercept is equal to −1/*K*_M_. (**D**) Comparison of the kinetic parameters between WT Mb and the reported carboxylesterases (PLE1 and PLE6) [21].

To determine the β-lactamase activity of Mb, we measured the hydrolysis rate of AMP catalyzed by WT Mb or F46C/L61C Mb in the absence or the presence of 20 eq. Im (Figure 5A). The results showed that the reaction rate of F46C/L61C Mb was significantly reduced by ~62.2% compared to that of WT Mb, due to the effect of bis-His heme coordination. 

Since both WT Mb and F46C/L61C Mb have the same protein surface, plain acid-base or direct catalysis by surface residues should contribute equally to the activity. Moreover, the presence of Im further reduced the reaction rate of both WT Mb and F46C/L61C Mb by 22~24%. This could be due to the direct coordination of Im to the heme iron, as shown previously by Im titration studies, although a full conversion to Im/His93 coordination required ~17.2 mM of Im [45].

To determine the kinetic parameters for the β-lactamase activity, we performed hydrolysis studies under various AMP concentrations and fit the data to the Michaelis–Menten equation (Figure 5B and Table 1) and the Lineweaver–Burk plot (Figure 5C), respectively. The results showed that WT Mb exhibited a *k*_cat_ value of ~404 min^−1^, which is ~2.9-fold higher than that of F46C/L61C Mb and is almost one-half of that reported for the New Delhi metallo-β-lactamase-1 (NDM-1) [47]. The *K*_M_ value of WT Mb for AMP is close to that reported for carboxylesterases (PLE1 and PLE6, [21]), whereas the reaction rate (*v*_max_) is about 2.3~3.0-times higher than that of the latter (Figure 5D).

Furthermore, WT Mb exhibited a catalytic proficiency ((*k*_cat_/*K*_M_)/*k*_uncat *_) of 6.25 × 10^10^, which is ~30,000-fold higher than that of an artificial metallo-β-lactamase reported previously [20]. The addition of Im decreased the *k*_cat_ value and increased the *K*_M_ value of WT Mb, resulting in a decrease in the catalytic proficiency by ~54%, which indicates that the heme center is responsible for the β-lactamase activity. This is further supported by observations for F46C/L61C Mb with reduced activity by adopting bis-His heme coordination.

These results suggest that WT Mb may serve as a potential β-lactamase, although the activity is lower than those of native metallo-β-lactamases [47]. It should be noted that there are Mb-like proteins, such as hemoglobin (Hb), in the blood that exist in the form of tetramers. Although β-lactamase activity has not been reported, Hb was found to catalyze the degradation of coenzyme A (CoA) by hydrolytic cleavage [48]. The β-lactamase activity of Mb might destroy lactam antibiotics and reduce the therapeutic effect; however, this requires further studies to investigate the biomedical relevance.

### 2.4. Effects of the Hydrolysis Product of AMP on the Growth of E. coli Cells

**Figure 6:**
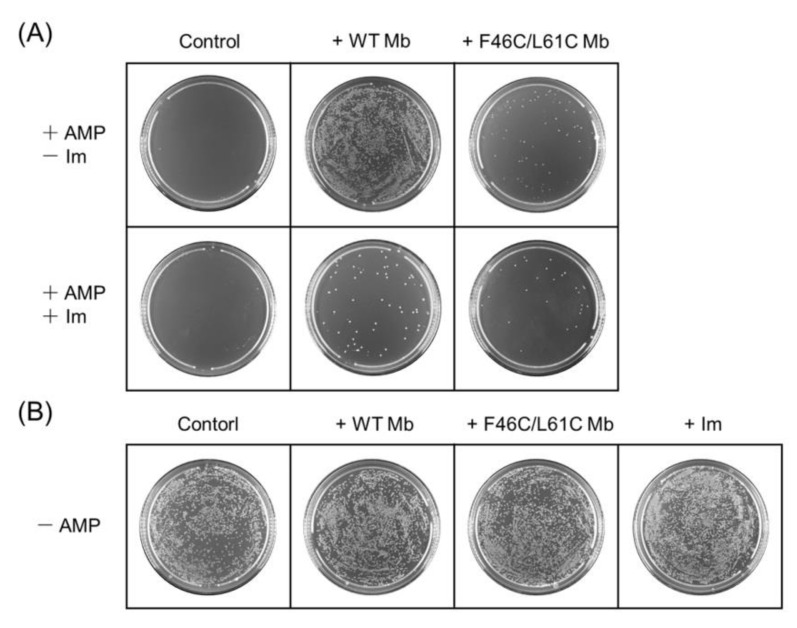
(**A**) Representative LB/agar plates with AMP (2 mM) and Mb (100 nM) in the absence (up) and presence (down) of Im (2 μM). (**B**) LB/agar plates of BL21(DE3) *E. coli* cells with or without WT Mb, F46C/L61C Mb and Im, respectively.

In order to evaluate the effect of AMP on the growth of *E. coli* cells upon its hydrolysis catalyzed by Mbs, we cultured the cells on LB/agar plates by adding the solution containing AMP or its hydrolysis product. As shown in Figure 6A, Im did not affect the activity of AMP, whereas the addition of AMP almost completely inhibited the growth of *E. coli* cells. For the plates with Mbs, *E. coli* cells grew unimpeded with the existence of AMP or its hydrolysates on the plate of WT Mb. One containing F46C/L61C Mb can be seen clearly, yet the number of colonies is significantly reduced compared with the former. 

Compared with the plate with ether WT Mb or F46C/L61C Mb, the presence of Im affected the activity of Mb and further reduced the colony’s number of *E. coli* cells. According to the control studies in Figure 6B, neither Mbs nor Im can inhibit the growth of *E. coli* cells. Thus, these results reinforce that the heme active site is indeed crucial for the activity and that the heme coordination state affects the β-lactamase activity of Mb.

## 3. Conclusions

To summarize, we found that, as an O_2_ carrier, Mb also exhibits β-lactamase activity by catalyzing the hydrolysis of ampicillin, although the activity is lower than the activity of native metallo-β-lactamases, and this was regulated by the heme coordination state, such as by bis-His or imidazole coordination. ESI-MS and HPLC studies suggested that Mb can cleave the β-lactam ring of AMP to inactivate it under mild conditions (pH 7.0). HPLC kinetic studies and *E. coli* cell growth experiments further revealed that the formation of a disulfide bond, as designed in F46C/L61C Mb, and direct Im coordination significantly inhibited the β-lactamase activity. These observations indicate that the heme active center is responsible for the β-lactamase activity of Mb. To the best of our knowledge, this is the first study showing that the β-lactamase of Mb might be biomedically relevant, which also suggests the potential of heme proteins acting as β-lactamases, thereby, broadening the diversity of their catalytic functions. 

## 4. Materials and Methods

### 4.1. Protein Preparation

Wild-type (WT) sperm whale Mb and F46C/L61C Mb were expressed in BL21(DE3) *E. coli* cells and purified as reported previously [43].

### 4.2. Mass Spectrometry Studies

The WT Mb or F46C/L61C Mb sample was desalted with a PD10 column and diluted with 0.1 M acetic acid (pH 3.0) to ~20 μM. The protein solution was mixed with 1% formic acid, which was transferred into the mass spectrometer chamber for measurement in positive mode. The data were analyzed using G2-XS QTOF mass spectrometry (Waters), and the multiple *m*/*z* peaks were transformed into the protein molecular weight by using the software MassLynx V4.1. AMP and its hydrolysis product, as catalyzed by WT Mb or F46C/L61C Mb, were measured in positive mode.

### 4.3. High-Performance Liquid Chromatography (HPLC) Studies

The mixture containing freshly prepared AMP and 20 nM WT Mb or F46C/L61C Mb in the absence and presence of 400 nM Im with a final volume of 2 mL was reacted at 25 °C. Injections were made each ~9 min for ~1 h, and the quantitative analysis was performed by monitoring the absorbance at 206 nm and integrating the peak areas. The retention times of AMP and the hydrolyzed product were ~3.2 and ~7.8 min, respectively, which were confirmed by ESI-MS. 

The mobile phase was a mixture of 20 mM potassium phosphate buffer and acetonitrile in a 1:7 (*v*/*v*) ratio with a flow rate 0.7 mL/min. The hydrolytic rates of the proteins were determined at various AMP concentrations. The data were fit to the Michaelis–Menten equation, *v* = *k*_cat_[E]_0_[S]/(*K*_M_ + [S]), where *v*, [E]_0_ and [S] are the rate, enzyme concentration and substrate concentration, respectively, and this yields the *k*_cat_ and *k*_cat_/*K*_M_ values.

### 4.4. Growth of E. coli Cells under the Hydrolysis of AMP

BL21(DE3) *E. coli* cells were grown on agar/Luria–Bertani (LB) plates with the mixture containing 2 mM freshly prepared AMP and 100 nM WT Mb or F46C/L61C Mb in the absence and presence of 2 μM Im (each mixture was incubated for 1 h in advance and incubated with E. coli cells for 30 min before coating the plates). For control studies, BL21(DE3) *E. coli* cells were mixed with AMP, WT Mb, F46C/L61C Mb or Im alone and grown under the same conditions.

## Figures and Tables

**Table 1:** Summary of the β-lactamase activities of WT Mb and F46C/L61C Mb (20 nM) with or without Im (400 nM) for the hydrolysis of AMP as measured in 20 mM potassium phosphate buffer (pH 7.0).

Proteins	*k*_cat_ (min^−1^)	*k*_cat_/*K*_M_ (min^−1^ M^−1^)	Rate Enhancement *k*_cat_/*k*_uncat_ *	Catalytic Proficiency (*k*_cat_/*K*_M_)/*k*_uncat_ *
WT Mb	404.38 ± 7.48	4.41 × 10^6^	5.73 × 10^6^	6.25 × 10^10^
WT Mb + Im	402.46 ± 32.89	2.04 × 10^6^	5.70 × 10^6^	2.89 × 10^10^
F46C/L61C Mb	141.27 ± 3.80	2.44 × 10^6^	2.01 × 10^6^	3.45 × 10^10^
F46C/L61C Mb + Im	95.49 ± 3.36	2.39 × 10^6^	1.35 × 10^6^	3.39 × 10^10^

* The background (uncatalyzed) rate constant for AMP hydrolysis was measured to be *k*_uncat_ = 7.06 × 10^−5^ min^−1^ (Figure 4A).

## Data Availability

The datasets for this manuscript can be obtained from the corresponding author upon reasonable request. The samples of the compounds and/or corresponding spectra are available from the authors.
